# Community health workers at the dawn of a new era: 5. Roles and tasks

**DOI:** 10.1186/s12961-021-00748-4

**Published:** 2021-10-12

**Authors:** Claire Glenton, Dena Javadi, Henry B. Perry

**Affiliations:** 1 Global Health Unit, Norwegian Knowledge Centre for the Health Services, Oslo, Norway; 2 TRS Centre for Rare Disorders, Sunnaas Hospital, Nesodden, Norway; 3 Department of Social and Behavioral Sciences, Harvard TH Chan School of Public Health, Boston, MA United States of America; 4 Department of International Health, Health Systems Program, Johns Hopkins Bloomberg School of Public Health, Baltimore, MD United States of America

**Keywords:** Community health worker, Health worker roles, Programme planning, Evidence-based guidance

## Abstract

**Background:**

This is the fifth of our 11-paper supplement on “Community Health Workers at the Dawn of a New Era.” When planning new community health worker (CHW) roles or expanding existing roles, programme planners need to analyse global and local research evidence and evidence-based guidance on the effectiveness and safety of relevant tasks performed by CHWs.

**Methods:**

In this paper, we explore key areas of consideration when selecting roles and tasks; present current knowledge regarding these issues; and suggest how decision-makers could consider these issues when assigning tasks in their setting. This paper draws on the chapter “Community Health Worker Roles and Tasks” in *Developing and Strengthening Community Health Worker Programs at Scale: A Reference Guide and Case Studies for Program Managers and Policymakers*, as well as on a recently published compendium of 29 case studies of national CHW programmes and on recently published literature pertaining to roles and tasks of CHWs.

**Results:**

This paper provides a list of questions that aim to help programme planners think about important issues when determining CHW roles and tasks in their setting. Planners need to assess whether the recommended roles and tasks are considered acceptable and appropriate by their target population and by the CHWs themselves and those who support them. Planners also need to think about the practical and organizational implications of each task for their particular setting with regard to training requirements, health systems support, work location, workload, and programme costs.

**Conclusion:**

When planning CHW roles and tasks, planners, programme implementers, and policy-makers should draw from global guidance and research evidence, but they also need to engage with the experiences, needs, and concerns of local communities and health workers. By drawing from both sources of information, they will stand a better chance of developing programmes that are effective in achieving their goals while remaining acceptable to those affected by them, feasible to implement, and sustainable over time.

**Table:** 

Key message box 1. Summary	
Key findings	
This article provides a list of 10 questions that can help programme planners think about important issues when determining CHW roles and tasks: • How effective and safe will it be to use CHWs to perform a specific task? • Are CHWs’ roles and tasks likely to be regarded as acceptable by CHWs and their clients and communities? • Are CHW roles and tasks considered relevant by the community? • Is there a good match between the CHWs and the roles and tasks expected of them? • How many tasks and activities should each CHW take on? • What is most feasible for the health system? • When and where will CHWs deliver each task and how much workload will it require? • What kind of skills and training will the CHW need when performing specific tasks? • What type of health system support will the CHW require when performing the task? • How much will it cost to use CHWs to perform the task?	
Key implications	
• When planning new CHW roles or expanding the roles of existing CHWs, programme planners need to base their decisions on global and local evidence and guidance • Planners need to consider the effectiveness and safety of relevant tasks performed by CHWs. They also need to assess whether the recommended CHW roles and tasks are considered acceptable and appropriate by their target population and by the CHWs themselves and those who support them. Finally, planners need to think about the practical and organizational implications of each task for their particular setting with regard to training requirements, health systems support, work location, workload, and programme costs.	

## Introduction

Large-scale community health worker (CHW) programmes may be at the threshold of a new era. This is indicated by the release of the WHO 2018 guidelines for optimizing the contribution of CHWs [1] and the World Health Assembly’s 2019 declaration of the importance of CHWs for achieving global health goals [2]. Instead of being seen as a temporary, second-class solution to problems that will gradually go away, CHW programmes are increasingly seen as an important health systems anywhere for the long term. Such programmes have long been a key element of many healthcare systems, particularly in low- and middle-income countries. However, the persistence of factors including poor access to basic services, a shortage of health workers, and a growing recognition of the need for task shifting has led to even greater interest in CHWs, including in developed countries. In addition, as healthcare trends change and new events occur, countries are turning to CHW programmes. CHWs are increasingly recognized as potential providers of services tied to noncommunicable diseases (NCDs) [3] such as hypertension [4], diabetes [5], cancer screening [6–8], and mental health [9]; as well as services tied to environmental health [10], digital health [11], and humanitarian events [12, 13]. This interest has also increased in the face of the coronavirus disease 2019 (COVID-19) pandemic [14]. In a recent article in *The Lancet*, a group of United Kingdom-based health systems researchers proposed the creation of a cadre of 100,000 CHWs initially as part of the United Kingdom’s COVID-19 response but potentially also as a long-term model of care [15]. The authors of the *Lancet* paper describe several tasks that CHWs could take on, including regular home visits to the elderly and other vulnerable groups, assessment of any health-related or social needs, early detection of illness, and assessment of the need for more care. In the final paper of this series [16] we provide additional perspectives on emerging roles for CHWs.

But what type of considerations should programme planners make when determining the roles and tasks of CHWs? In this article, we will discuss the specific roles and tasks that CHWs currently have and present a list of questions that planners and programme managers should consider. These include questions about the effectiveness and safety of using CHWs to perform specific tasks; the acceptability of different options among CHWs, community members, and other stakeholders; and the feasibility of these options, for instance, with regard to workload, training requirements, health system support, and costs. The article is an update of the chapter “Community Health Worker Roles and Tasks” in *Developing and Strengthening Community Health Worker Programs at Scale: A Reference Guide and Case Studies for Program Managers and Policymakers* [17] (referred to here as the CHW Reference Guide) [18]. We also draw on the recently published compendium of 29 case studies of national CHW programmes [19] as well as recently published literature pertaining to roles and tasks of CHWs.

CHW programmes have been the focus of much research over the last few decades, and this evidence can offer important insights to decision-makers and programme planners about appropriate roles and tasks. At the same time, decisions about CHW roles and tasks are highly contextual. The goal of this paper is therefore not to offer a prescriptive method for assigning roles and tasks. Instead, this paper seeks to: (1) explore key areas of consideration when selecting roles and tasks; (2) present current knowledge regarding these issues; (3) and suggest how decision-makers could consider these issues when assigning tasks in their setting.

## What kind of roles and tasks do CHWs carry out at present?

**Table:** 

Key message box 1	
CHWs have traditionally focused on roles and tasks tied to maternal and child health, but their roles and tasks are expanding	

CHW programmes have traditionally focused on roles and tasks tied to maternal and child health. Increasingly, however, CHW programmes also commonly provide services tied to HIV and tuberculosis (TB), and more recently, NCDs, care of the elderly, mental healthcare, and palliative care. Within these topics, the tasks that CHWs take on can be organized into several different categories: health promotion and preventive care, community mobilization, treatment/clinical curative care [20], and epidemographic surveillance [21]. There are other categories that could be included here, such as the role of *liberator* mentioned in Paper 1 [22] (encompassing such roles a social change agent, builder of social capital, and so forth), but to address these issues is complex and goes beyond the scope of this paper.

### Community mobilization

Many CHWs act as community mobilizers, initiating activities such as the digging of latrines, the identification of clean water sources, and the organization of nutrition and sanitation days. In some programmes, CHWs help to mobilize the community so that when a government vaccinator comes to the community, those who need a vaccination will be there to receive it.

### Health promotion

Perhaps the most common role taken on by CHWs is that of health promoter. Here, the CHW provides information and counselling with the aim of encouraging particular behaviours, including health service utilization. Typical tasks include the promotion of breastfeeding and child nutrition, family planning, immunization, and other behaviours linked to mother and child health, as well as the promotion of safe sex and encouragement of HIV testing. In addition, CHWs are sometimes used to promote awareness about social welfare issues such as domestic violence or alcohol and drug abuse. More recently, the promotion of breast cancer screening has been included in some programmes.

### Provision of preventive services

CHWs play important roles in prevention. In addition to educating families about immunizations and mobilizing communities to attend immunization sessions, CHWs in a number of countries administer immunizations, including oral polio vaccine. Other preventive services provided by CHWs include administration of vitamin A and other vitamins as well as deworming medication. CHWs provide family planning services—not only the distribution of birth control pills and condoms but also the provision of injectable contraceptives and even in some cases, the insertion of long-acting subcutaneous contraceptive implants and intrauterine devices.

CHWs also commonly provide preventive healthcare services by distributing commodities such as those mentioned above as well as insecticide-treated bed nets (for prevention of malaria) and iron/folate tablets for pregnant women. In some settings, CHWs dispense misoprostol tablets to pregnant women in their third trimester who plan to give birth at home to take immediately after the birth of the child (and before the delivery of the placenta) in order to promote uterine contraction and reduce the risk of postpartum haemorrhage. In some settings, CHWs also dispense chlorhexidine antiseptic to be applied to the newborn umbilical cord stump to reduce the risk of neonatal sepsis.

### Provision of clinical services

Another role of growing importance involves the diagnosis and management of illness. This commonly includes tasks such as recognition of danger signs in children and pregnant women and increasingly the diagnosis and management of common childhood illnesses. CHWs in some settings also provide assistance to women during labour and delivery, although this role is most commonly limited to providing support to the mother in the presence of a skilled birth attendant. CHWs sometimes also carry out home visits to newborns to provide support to mothers in carrying out healthy practices for the care of their newborn, identifying newborns with possible complications, and initiating treatment and/or referral.

Many CHW programmes also support the identification of persons with symptoms suggestive of TB, facilitation of collection of sputum in the home for laboratory testing, and support for daily treatment at home for those who test positive, including direct observation of pill taking. Similarly, many programmes engage CHWs in HIV programming—not only in promotional activities but also in actually carrying out voluntary counselling and testing in the home as well as providing adherence support for those on antiretroviral medication. In many countries now, CHWs have also been trained to diagnose (often using rapid diagnostic tests that can be performed in the home) and treat malaria, not only in children but also in those older than 5 years of age.

### Epidemographic surveillance and record keeping

Other tasks included in some CHW programmes are routine visitation of all households, mapping and registration of all households as part of a census-taking and/or census-updating activity, registration of vital events, and surveillance for infectious diseases such as acute flaccid paralysis for potential cases of polio, measles, neonatal tetanus, TB, Ebola and COVID.

### Integration of roles

In most settings, CHWs deliver tasks that cut across these categories. For instance, a common CHW role is to provide services under the rubric of integrated community case management (iCCM) of childhood illness. Here, in addition to tasks tied to promotion and prevention, CHWs are trained to manage common but potentially life-threatening childhood illnesses such as uncomplicated pneumonia, diarrhoea, malaria, and undernutrition, as well as to refer children with serious danger signs to a health facility. Similarly, CHWs in some settings take on a range of activities tied to palliative care, including raising awareness about and identifying individuals requiring palliative care in the community, assistance with pain management, holistic home-based care and visitation, and provision of psychological support and spiritual guidance.

Table 1 presents a summary of the roles of CHWs as described in the recent compendium of 29 case studies entitled *Health for the People: National Community Health Worker Programs from Afghanistan to Zimbabwe* [19].

**Table 1:** Summary of roles and tasks performed by CHWs in 29 national case studies [19]

Role	Examples of related tasks	Examples of CHW programmes where this is carried out
Community mobilization	Build community awareness for immunization	BRAC’s *Shasthya Shebikas* mobilize mothers to bring their children to outreach sessions when visiting immunization teams come to the community India’s ASHA workers and Anganwadi workers promote awareness about and participation in national polio immunization days as part of the Global Polio Eradication Initiative CHWs across Africa promote awareness in child health days, at which time immunizations are provided, vitamin A and deworming medicines are given, and commodities such as bed nets are distributed
Health promotion (including health education)	Promotion of healthy household behaviours	Ethiopia’s Women’s Development Army volunteers promote healthy household behaviours Pakistan’s LHWs provide nutrition education
	Family planning education: counselling about birth spacing, family planning, and contraceptives	Afghanistan’s CHWs offer a package of services including health promotion of family planning Bangladesh’s family welfare assistants (FWAs) and BRAC’s *Shasthya Shebikas* in Bangladesh promote family planning and provide information on how to access services
	Promotion of ANC and PNC	BRAC’s *Shasthya Shebikas* in Bangladesh; Mozambique’s APEs, Sierra Leone’s CHWs, Zimbabwe’s CHWs
	Promote the utilization of facility-based delivery	India’s ASHAs receive an incentive payment for bringing a woman to a facility for delivery
	Nutrition education: counselling about infant and child feeding (including breastfeeding)	Anganwadi workers in India, *Kaders* in Indonesia, CHWs in Madagascar
	Promotion of the importance of clean water, sanitation, and hygiene	CHVs in Kenya, CHWs in Sierra Leone
Provision of preventive services	Provision of vitamin A supplementation; oral polio immunization; injectable immunizations; deworming medication; and iron, folate and other vitamins	*Shasthya Shebikas* and *Shasthya Kormis* in Bangladesh provide vitamin A supplementation and deworming medication Malawi’s HSAs administer vitamin A supplementation and deworming medication, and they give immunizations as well Nepal’s FCHVs provide deworming medication, vitamin A supplementation, and polio immunization
	Distribute of condoms, insecticide-treated bed nets, oral rehydration salt (ORS) packets	*ASCs* [community health workers]) in Niger distribute insecticide-treated bed nets Health assistants in Bangladesh distribute ORS packets
Provision of clinical services	Identification of pregnant women and children with warning or danger signs for which treatment should be sought	CHOs in Ghana refer patients needing higher-level care and follow up on those patients who had been referred VHWs in Nigeria refer pregnant women to health facilities
	Management of childhood illness (identification of cases, management of uncomplicated cases, referral of cases with complications)	Integrated management of childhood illness is a common role across CHW programmes, including Ghana’s CHOs, Afghanistan’s CHWs, Niger’s *ASCs*, and Zimbabwe’s VHWs
	Provision of ANC and PNC	Pakistan’s LHWs coordinate ANC/PNC services
	Provision of basic curative services	Ethiopia’s HEWs provide basic curative services for selected illnesses, including support to people with chronic illness (e.g., HIV/AIDS patients) Zimbabwe’s CHAs provide basic curative care and refer patients to facilities for further treatment, documenting referrals in community health registers Iran’s *Behvarzs* and *Moraghebe-salamats* provide management of NCDs such as diabetes and hypertension as well as oral healthcare Programme assistants working with BRAC in Bangladesh evaluate people with eye problems and provide eyeglasses for older patients with presbyopia
	Provision of clinical family planning services: provision of birth control pills, injectable contraceptives, and sometimes placing longer-acting contraceptives such as IUDs and subcutaneous implants	Ethiopia’s HEWs provide a full range of family planning services, including provision of longer-acting contraceptives Ghana’s CHOs provide maternal and reproductive health services as well as management of minor ailments
	Identification of persons with symptoms of TB and referral for further evaluation (or collection of sputum for examination at a TB programme laboratory); provision of directly observed treatment for patients diagnosed with TB	Training packages in Afghanistan have expanded to provide DOT for TB Bangladesh’s *Shasthya Shebikas* identify suspected TB cases, collect sputum for diagnosis, and provide DOT to diagnosed patients Rwanda’s CHWs support diagnosis and treatment of TB patients
	Provision of first aid for injuries/accidents	Kenya’s CHVs treat common ailments and minor injuries with guidance from the community health extension workers
	Provision of mental health services	Afghanistan’s CHWs provide referral for mental health support, but they are trained to provide direct service provision for those with mental health problems and physical disabilities Indonesia’s *Kaders* are expanding their roles to include mental health counselling Iran’s *Behvarz*s and *Moraghebe-salamats* are trained to manage mental disorders Liberia’s CHAs are trained to identify mental health problems and to identify side effects of mental health medications
Epidemographic surveillance and record-keeping	Household visits for surveillance: maintaining health records and vital event registration, and surveillance of emerging diseases and of disease prevalence	Brazil’s CHAs maintain household registers and visit households monthly Guatemala’s mobile health teams maintain a community census and conduct epidemiological monitoring India’s ASHA workers carry out malaria case identification and screening for NCDs Sierra Leone’s CHWs carry out community-based disease surveillance, making quarterly household visits to promote preventive behaviours, and collect essential demographic information

*ANC* antenatal care, *APE* *Agents Polivalente Elementare*, *ASC* Agent de Sante Communautaire (Niger), *ASHA* accredited social health activist, *BRAC* Building Resources Across Communities, *CHA* community health agent (in Brazil), *CHA* community health assistant (in Liberia), *CHO* community health officer, *CHV* community health volunteer, *CHW* community health worker, *DOT* directly observed therapy, *FCHV* female community health volunteer, *HSA* health surveillance assistant, *HEW* health extension worker, *LHW* lady health worker, *NCD* noncommunicable disease, *ORS* oral rehydration salts, *PNC* postnatal care, *VHW* village health worker

### Creation of trust and social support

The abovementioned roles that CHWs can perform are instrumental, meaning that they serve to assist health systems in achieving specific aims or policies. However, there are also other important functions that CHWs perform in the process of carrying out these instrumental roles. As Schaaf et al. emphasize, “While CHWs are often conceptualized as cogs in a mechanistic health delivery system, at the end of the day, CHWs are people embedded in families, communities, and the health system” [23] [p. 1].

One of the CHWs’ social functions is building trust with the community. Trust from the community is a critical asset that health systems need and is built through “cooperation, continuous and open communication, personal and professional motivation and empathy” [24]. Through their personal contact with the community, CHWs can build trust in the community with the health system and with the CHW herself, as noted, for example, by accredited social health activist (ASHA) workers in India [25].

Another social function provided by CHWs is the provision of emotional and social support. This type of support can be particularly important for those who are lonely (for instance, elderly people with limited social interaction) and those who are stigmatized for some reason. A systematic review of CHW programmes showed that in programmes from high-income countries in particular, mothers with young children emphasized the importance of CHWs as a source of emotional and social support [26]. These types of functions have been highlighted among CHWs in Brazil (community health agents) who visit all the homes in their catchment area on a monthly basis [27]. This provides a “space for dialogue, due to the time spent together, the close relationship and the trust for care” and creates “affective bonds … [in which] the practices of the CHW are guided mainly by empathy and affective listening” [27] [pp. 667–8].

A further social function that CHWs provide is the simple act of providing assistance to someone in need. The examples of CHWs assisting patients and families in need because of a medical problem are legion. They include, among other things, helping patients reach a health facility during an emergency or simple acts of kindness to someone during a personal crisis. For instance, the contributions of female community health volunteers following the 2015 earthquake in Nepal in providing the first wave of assistance has been widely noted [28] as well as the contributions of community health agents in Brazil following disasters [13].

## What key questions do programme planners need to consider when selecting CHW roles and tasks?

While there is enthusiasm for the important contributions that CHWs can make to expanding the availability of healthcare and improving population health, there also needs to be a proper understanding and appreciation of the potential stressors and strains that the role of CHW carries with it. Table 2 provides a snapshot of some of the stressors and strains that have been reported by CHWs in various countries. CHW roles and the enabling environment to effectively carry out tasks are affected by various components of the health system. For example, access to supplies, medicine, equipment, infrastructure, and transportation are external factors that affect the ability to deliver services. Meanwhile, adequate pay and working conditions as well as access to supportive supervision and up-to-date training are factors that can affect intrinsic motivation. As enthusiasm waxes for strengthening CHW programmes and for expanding their roles beyond maternal and child health, it is important to carefully consider how we can design and operate programmes that create a supportive environment and avoid as many stressors and strains as possible. Here, we raise a number of questions that are worthy of consideration.

**Table 2:** Examples of stressors and challenges faced by CHWs

Type of CHW	Stressors
Lady health workers (Pakistan) [29]	Lack of adequate training to communicate effectively with families Lack of skills to perform the tasks required Low socioeconomic status (can cause lack of respect and harassment) Need to travel long distances by foot Stock-outs of medical supplies Low salary Lack of a career structure
ASHA workers (India) [30]	Violent attacks by men Lack of respect from superiors due to families’ standing and level of education Low salary (this has psychosocial implications such as feeling alienated and undervalued for the disproportionate burden they now bear, particularly in light of their new role in India’s battle against COVID-19) [30]
HEWs (Ethiopia) [31]	Lack of community trust in the services and the products HEWs provide
Village health team members (Uganda) [32–34]	Lack of trust from other health-care providers who view CHWs as not appropriately trained and a government ploy for control Conflicts with higher-level staff Stock-outs of medicines and equipment Lack of respect from community members and government officials (mistrust and stigma can lead to emotional trauma and depressive symptoms, as seen during the Ebola and Marburg virus outbreaks)
CHWs (Tanzania) [35]	Stock-outs of medicines and supplies
CHEWs (Nigeria) [36]	Lack of training for the reality of the job
Health surveillance assistants (Malawi) [37]	Lack of adequate training Inadequate supervision Work overload due to the extensive needs of the community Low pay
Women’s Development Army volunteers (Ethiopia) [38]	Psychosocial challenges, including becoming the subject of gossip
CHWs in Papua New Guinea [39]	Young female CHWs feel unsafe and afraid because of abuse from young men, violent assaults, and accusations

*ASHA* accredited social health activist, *HEWs* health extension workers, *CHEWs* community health extension workers

### How effective and safe will it be to use CHWs to perform a specific task?

**Table:** 

Key message box 2	
Programme planners need to consider the effectiveness and safety of tasks when provided by CHWs	

When planning healthcare services, decision-makers need to consider the effectiveness and safety of specific interventions. CHW programme planners also need to consider these issues. While we know that interventions such as newborn resuscitation, oxytocin, and misoprostol for postpartum haemorrhage and antibiotics for neonatal sepsis [40] can improve health and save lives, how do we decide which interventions can be delivered by CHWs?

When making these decisions, programme planners should explore current evidence and evidence-based guidance. The recently released WHO guidance for CHWs contains guidelines and manuals related to the technical aspect of intervention implementation for different topics including maternal, newborn, and child health; reproductive health; HIV and TB; response to epidemics and humanitarian emergencies; management of stress; and response to intimate partner violence and sexual violence against women [1] [p. 100–102]. WHO guidance is now also available for community-based services in the midst of the COVID-19 pandemic [14].

This guidance is informed by a growing body of evidence that concludes that the promotion of certain healthcare behaviours and services by CHWs, such as the promotion and support of breastfeeding and childhood immunization, can lead to significant health improvements [41]. When it comes to the more curative tasks, there are, however, many evidence gaps [41–43]. For this reason, the WHO recommended in 2012 that a number of tasks should be performed by CHWs only in the context of either monitoring and evaluation or rigorous research [40]. In other words, policy-makers and programme planners have been encouraged to pilot curative interventions and to conduct a rigorous assessment of their effectiveness in their setting. Table 3 presents a list of interventions that were listed in the 2012 WHO guidance that CHWs could provide for maternal and newborn care [40]. Similar lists could be developed that are more current and that apply to the aspects of CHW work other than tasks related to maternal and newborn care.

**Table 3:** Current WHO recommendations concerning the use of CHWs for maternal and newborn health [40]

**Recommended interventions to be provided by CHWs for maternal and newborn health:**
• Promotion of the uptake of health-related behaviours and healthcare services for maternal, HIV, family planning, and neonatal health, including: • Promotion of appropriate care-seeking behaviour and antenatal care during pregnancy • Promotion of companionship during labour • Promotion of sleeping under insecticide-treated bed nets during pregnancy • Promotion of birth preparedness • Promotion of skilled care for childbirth • Promotion of adequate nutrition and iron and folate supplements during pregnancy • Promotion of reproductive health and family planning • Promotion of HIV testing during pregnancy • Promotion of exclusive breastfeeding • Promotion of postpartum care • Promotion of immunization according to national guidelines • Promotion of kangaroo mother care for low birth weight infants • Promotion of basic newborn care and care of low birth weight infants • Administration of misoprostol to prevent postpartum haemorrhage • Provision of continuous support for women during labour in the presence of a skilled birth attendant
**Intervention recommended only in the context of monitoring and evaluation:** • Distribution of oral supplements to pregnant women (e.g., calcium supplementation for women living in areas with known low levels of calcium intake; routine iron and folate supplementation; vitamin A supplementation for pregnant women living in areas where severe vitamin A deficiency is a serious public health problem) • Intermittent presumptive therapy for malaria for pregnant women living in endemic areas • Provision of injectable contraceptives
**Interventions recommended only in the context of rigorous research:** • Oxytocin administration to prevent postpartum haemorrhage—standard syringe • Oxytocin administration to treat postpartum haemorrhage—standard syringe • Oxytocin administration to prevent postpartum haemorrhage—CPAD • Oxytocin administration to treat postpartum haemorrhage—CPAD • Misoprostol administration to treat postpartum haemorrhage • Low-dose aspirin distribution to pregnant women at high risk of pre-eclampsia/eclampsia • Puerperal sepsis management with intramuscular antibiotics—standard syringe • Puerperal sepsis management with oral antibiotics • Puerperal sepsis management with intramuscular antibiotics—CPAD • Initiation of kangaroo mother care for low birth weight infants • Maintenance of kangaroo mother care for low birth weight infants • Injectable antibiotics for neonatal sepsis—standard syringe • Antibiotics for neonatal sepsis—CPAD • Neonatal resuscitation • Insertion and removal of contraceptive implants
**WHO does not recommend using CHWs for the insertion and removal of intrauterine devices**

*CPAD* compact, prefilled, auto-disabled injection device

### Are CHWs’ roles and tasks likely to be regarded as acceptable by CHWs and their clients and communities?

**Table:** 

Key message box 3	
Programme planners need to consider the acceptability and relevance of CHW roles and tasks to the CHWs themselves and to the community	

In addition to effectiveness and safety, programme planners need to assess whether potential CHW roles and tasks are considered acceptable and appropriate by the CHWs themselves, their target population, and the wider community. Attempts to introduce roles and tasks that do not find support among these groups are unlikely to be successful or sustainable.

#### Are CHW roles and tasks considered relevant by the community?

Problems can occur when CHWs are confronted with issues during their daily activities that are considered more important than the issues that they have been trained to address. For example, in communities where members suffer from a number of health problems not addressed by the programme and where they have poor access to other healthcare services, CHWs may frequently be approached about issues that are outside their scope of training. CHWs may also find themselves spending a lot of their time on tasks that feel less pressing, such as documentation or surveillance tasks [44] or may be confronted with related problems of the clients such as lack of housing, food insecurity, alcohol abuse, and social and domestic violence. This issue is a particular challenge for CHWs whose scope of practice is defined as health-related only.

A mismatch between the needs and wishes of the community and the services CHWs have to offer are likely to influence recipient satisfaction and uptake of services and can lead to feelings of frustration and impotence among the CHWs themselves [26]. Some CHWs may find it particularly frustrating to deliver promotional services only and may want to offer ‘real healthcare’, such as medicines and immunizations [26]. The delivery of services that are valued by the community and by the CHWs, on the other hand, can increase uptake of these services as well as the CHW’s legitimacy and motivation. A recent six-country study concerned with four dimensions of CHW empowerment (meaningfulness, competence, self-determination, and impact) reported that the opportunity to perform meaningful and impactful work could give CHWs a sense of empowerment. However, a lack of control over their work environment and a sense of being unsupported, underappreciated, and undervalued could lead to feelings of frustration and powerlessness [45]. The authors propose the CHW programmes should take these issues seriously in order to improve CHW performance and help them become agents of social change.

The involvement of community members and CHWs in programme planning is critical to ensure that tasks are seen as relevant and useful. (This issue is also addressed in more detail in Paper 9 in this series [46].) In Nicaragua, the tasks of the CHW were extended to include curative healthcare. This led to an increase in CHW motivation and community respect and satisfaction [47]. However, any transition from promotional to curative tasks can also represent a double-edged sword, as it could leave CHWs vulnerable to blame if things go wrong [48]. CHWs offering services that can be perceived as harmful may be in particular need of visible support from community structures and health facilities. The introduction of more complex tasks could also lead programmes to favour CHWs with higher levels of education. This could, in turn, hinder the selection of CHWs from the local community, thus undermining the potential advantages of local recruitment (see below).

Community involvement may lead to a broader scope of practice for the CHW. And as discussed in more detail below, WHO’s 2018 support of *generalist* CHWs was driven by the argument that CHWs with a broader set of skills could better respond to peoples’ needs for services [1]. Community involvement may also lead to more attention given to activities that may be outside the health sector, such as awareness raising and prevention of domestic violence or the establishment of microcredit systems or gardening projects. This more holistic approach may be regarded as more satisfying and relevant to the CHW and the community. However, it may also require a more complex support system because of the needs for training, supervision, and supplies from sources outside the health sector and may also lead to a stretch of responsibilities and duties beyond what can reasonably be expected. Other factors, including the size of the catchment area and the time the CHW is expected to work also play a role when attempting to avoid work overload. The closer integration of CHWs into the primary healthcare team, as has been achieved in Brazil [49], can potentially address some of these issues and can allow more role expansion and flexibility when adjustments are required, as is the case with the current COVID-19 pandemic. Stronger community involvement could also provide stronger local support for CHWs. Where CHW programmes are decentralized, funding and programme planning and support could be managed more at a local level, making it possible to tailor CHW roles and tasks to local needs in particular geographic and ethnic areas.

#### Is there a good match between the CHWs and the roles and tasks expected of them?

The acceptability of the tasks provided by CHWs is also likely to be influenced by the type of CHW who performs them. CHW characteristics, such as gender, age, and life experience, and ties to the community can all play a role here. For instance, one study from Uganda illustrates how men were more comfortable discussing sexual and reproductive concerns with male CHWs, while women were more likely to disclose pregnancies to female CHWs [50]. In other settings, CHW programmes may promote the selection of young unmarried women to serve women of reproductive age, only to find that the young women need to be accompanied by older women as they do their home visits to provide credibility. CHWs’ place of residence and closeness to recipients may also influence acceptance. One advantage of selecting CHWs from the communities they serve is that local CHWs are more likely to possess relevant cultural competence, making it more likely that they understand and acknowledge local knowledge and attitudes about health and illness. Recipients may also prefer to receive services from CHWs they know well and trust. On the other hand, they may not want services to be delivered by close neighbours if these services are regarded as sensitive, such as the promotion of sexual and reproductive health.

Gender issues can also affect CHWs’ freedom of movement and the amount of time they have available, which again can impact on the acceptability and feasibility of certain tasks. For example, in Afghanistan, women are often not allowed to venture from their home unattended, while in Tanzania, home visits from male CHWs can be viewed as potentially being for ulterior or adulterous motives. To solve this issue, Afghanistan CHWs often work in pairs of women and men, and are frequently spouses or close family members [51]. Similarly, in Tanzania, male–female pairs help to negotiate some of these issues.

In Uganda, male CHWs’ access to motorcycles enables them to assist patients more quickly with referrals than female CHWs [52]. Female CHWs may also face time constraints because of traditional family duties and roles. For instance, in Mozambique, mothers, and particularly single mothers, may find it difficult to leave their family responsibilities for work and training [53].

These considerations are context-specific, and a general understanding of gender roles and expectations in the community is critical when selecting roles and tasks that are acceptable within the community. Programme planners and managers should approach this issue with care to ensure that empowerment and change happens in a way that can be integrated into the society rather than quickly rejected, leading to a weakening of CHW programmes.

### How many tasks and activities should each CHW take on?

Programme planners also need to think about the scope of the CHW’s role, whether he or she should have few but specific tasks or a broad repertoire of responsibilities. A related issue is whether each community should be offered different types of CHWs, each with his or her own specialty, or whether they should have access to one *generalist* CHW [54]. The 2018 *WHO guideline on health policy and system support to optimize community health worker programmes* [1] underlines that there is evidence to support both approaches. However, while acknowledging that some situations and settings may require specialized CHWs, the guideline argues that CHWs with a broader set of skills enables them to better respond to peoples’ needs for services. On this basis, the Guideline recommends that CHWs at the frontline of service delivery are trained to deliver a range of priority primary healthcare services, while CHWs with more selective and specific tasks can play a complementary role when required.

We have many examples of specialist CHWs financed by vertical programmes for family planning, HIV, malaria, TB, among others. In some settings, these vertical programmes have become integrated into a common primary healthcare platform through a professionalized, well-trained generalist CHW, as Ethiopia has done with its health extension workers (HEWs). The HEWs are responsible for providing not only general care but also family planning services, immunizations, screening and treatment for HIV and TB, among other specialized functions. There is a possibility that the growing numbers of tasks that HEWs perform could lead to role overload, especially when responsibilities for NCDs are added. Thus, the question then becomes whether to add more generalist HEWs to avoid overload and burnout or to support them with specialist CHWs who provide a narrower range of services.

One growing approach is for CHWs to work as a dual-cadre (or a two-tiered system) in which there is a more highly trained, usually salaried CHW working in tandem with a group of lesser-trained often volunteer CHWs who serve a small number of households. Ethiopia, Malawi, Mali, and Niger are examples [55, 56]. This makes it possible to distribute more tasks within the same catchment area. In Bangladesh, Building Resources Across Communities (BRAC) now has a specialized CHW called a programme assistant who travels from village to village and visits each village every 1–2 months to screen for hypertension, treat common eye diseases, and provide eyeglasses for older people with presbyopia (Morseda Chowdhury, personal communication, July 2020). There is one programme assistant for every 50–60 senior-level BRAC CHWs (*Shasthya Kormis),* and there is one *Shasthya Kormi* for every 10 lower-level BRAC CHWs (*Shasthya Shebikas*) [57].

It may also make sense to split some tasks between male and female CHWs according to what is most appropriate from a gender perspective. The establishment of male–female CHW pairs may also be helpful in settings where it is not safe or socially acceptable for women to travel alone. In Rwanda, there is within each village three specialized CHWs: there is a male/female pair (called *Binômes*) and one female *Animatrice de Santé Maternelle* (ASM). The female *Binôme* handles mostly maternal and child activities, while the male *Binôme* focuses on TB, malaria, and NCDs. The ASM focuses on pre- and postnatal care and family planning [58].

However tasks are distributed, both CHWs and target populations need to be involved in these decisions to help ensure that the correct balance has been achieved.

### What is most feasible for the health system?

**Table:** 

Key message box 4	
Programme planners need to consider the practical implications of different CHW roles and tasks	

In addition to the recipients’ and CHWs’ views about the breadth of tasks, programme planners also need to consider the practical implications of this decision for the health system. For example, CHWs who are expected to deliver a wide range of tasks will require more training and supervision than CHWs with fewer tasks. This decision also has implications for CHW payment and other incentives, as more tasks may lead to longer working hours, and CHWs can reasonably expect some form of acknowledgement for additional training and responsibilities. In contrast, it may be more efficient to train, supervise, and support a fewer number of *generalist* CHWs than to have the same number of tasks delivered by a greater number of *specialist* CHWs. Decisions regarding the number of tasks a CHW should have are also closely related to decisions regarding when and where each task will be performed and the workload each task entails. This issue is discussed further below.

### When and where will CHWs deliver each task and how much workload will it require?

Programme planners also need to think about when and where each task can or should be delivered by the CHW and the amount of work anticipated for the CHWs and their supervisors. These factors will have important implications, including the amount of flexibility and influence a CHW has over his or her workday, the appropriate catchment area, suitable incentives, and the opportunity to keep skills up to date. Programme planners will need to consider the need for transportation, safety measures, and the CHW’s freedom of movement.

The level of influence and flexibility a CHW has regarding *when* and *where* a task is performed can vary considerably. Some tasks, such as certain promotional tasks, can often be done in between a CHW’s other tasks, at his or her own convenience, and the CHW may also have a lot of flexibility regarding where the task can be done. For example, some CHWs may choose to use ad hoc opportunities and chance meetings, such as social or community events, to deliver certain promotional services. For other tasks, the CHW may have little influence on when and where they perform the task or how long it will take to complete the task. These include tasks such as continuous support during labour or other childbirth- and newborn-related tasks. It may also be necessary or preferable to perform other tasks inside the recipient’s home, while some tasks may need to be performed in clinics where CHWs can access supplies or can obtain needed supervision by higher-level health professionals.

When determining where tasks are performed, it is also important to assess what the target population regards as appropriate. For example, the extent to which home visits are socially acceptable will vary across settings and tasks.

CHWs with large workloads are likely to need more incentives than CHWs with lighter workloads. Demands for incentives may also be influenced by the amount of influence the CHW has over his or her working day. Tasks that can be performed within ordinary working hours may require fewer incentives than tasks that need to be performed in response to immediate needs that can occur at night or on weekends, such as childbirth-related tasks. Tasks that can be done at a time of the CHW’s choosing may be particularly appropriate for volunteer CHWs, as this flexibility makes it easier to combine with family and other responsibilities (see Paper 8 on motivation and remuneration [59]). From a programme planner’s point of view, however, it is reasonable to expect less from volunteers who work within the constraints of their own daily lives than from salaried CHWs.

Programme planners also need to think about when and where CHWs should deliver each task and the amount of work anticipated for CHWs and their supervisors. These decisions will have implications for the flexibility and control CHWs have over their workday, transportation and safety issues, appropriate catchment areas, suitable incentives, and the opportunity to keep skills up to date.

If the task requires the CHW to move around the catchment area, planners will need to consider the need for transportation. In some settings, CHWs travelling around the community or making home visits can be exposed to violence [26], so safety issues need to be carefully considered. These considerations may be particularly important if the task requires the CHW to travel at night. Suggested solutions include being accompanied by another individual, forming a working pair, and having access to mobile phones.

Different tasks also imply different workloads and catchment areas. Some tasks need to be delivered frequently or to large numbers of people. Therefore, the size of the CHW’s catchment area may need to be relatively small. Some tasks occur infrequently, such as immunization campaigns, or they target health conditions that are relatively rare. In these situations, it may seem reasonable to give CHWs a larger catchment area. However, large catchment areas imply that the CHW needs to cover longer distances, with implications for transportation needs. In addition, when catchment areas are too large, CHWs may spend too much time getting to the client or spending time on travel only to find that the client is absent [60]. Another challenge for tasks targeting health conditions that are relatively rare is the issue of quality of care as CHWs should ideally deliver tasks frequently enough to keep their skills up to date.

### What kind of skills and training will the CHW need when performing specific tasks?

Programme planners also need to think about the type of skills and training that CHWs will need to perform these tasks. When assessing these issues, programme planners may want to think about the following aspects:

- Is the task complicated to perform?
- Does the CHW need to tailor the task to the needs and circumstances of the individual recipient and the local context?
- Does the CHW need to make a complex diagnosis before performing the task?
- Does the CHW need to know how to deal with adverse effects or complications?

If the answer is “yes” to any of these questions, the task is likely to require more skills and training. Some tasks, such as the routine distribution of iron/folate supplements to pregnant women, are simple to perform, require little or no tailoring or diagnosis, and little knowledge about associated complications. Training may therefore be relatively short. Other tasks, such as training caregivers in the use of kangaroo mother care, are also relatively simple procedures to teach with few components. But, because in this case CHWs also need to have the skills to detect which infants need additional care and referral, training may be longer. Having well-developed, step-by-step instructions (algorithms), which can be paper-based or digital, can, to a certain extent, ease the requirements made of the CHW by providing the CHW with an additional form of support during decision-making (see Paper 6 on training [61]).

Promotional tasks may be regarded as simpler to perform than curative tasks. However, in a number of studies, CHWs have emphasized the importance of training in promotional and counselling skills, viewing health communication as a complex task for which they often feel unprepared [26]. For example, when promoting family planning methods or HIV testing, CHWs may need to respond to complex questions and concerns and may also experience socially challenging situations. The role of community organizer can also be a challenging one as it is likely to involve high degrees of tailoring and the ability to organize and mobilize groups of people and lead them in problem-solving activities.

### What type of health system support will the CHW require when performing the task?

Another practical implication that needs to be determined involves the level of healthcare system support required for each task. Some tasks can be performed by the CHW alone and with very little support from the rest of the healthcare system. For most tasks, however, successful delivery depends on a well-functioning and responsive health system.

Health system support may primarily involve supervision, typically from facility-based health workers. For example, Nepalese CHWs who identified young infants with possible severe bacterial infections were trained to administer gentamicin, but only if they were receiving regular supervision and observation from facility-based staff [62]. For this to work, CHWs need efficient ways of communicating with other health workers, such as access to transport or reliable mobile phone systems. [63]. CHWs can also receive supervision through peer support, for instance, by working together in teams or in pairs. CHWs in some studies have called for the opportunity to meet regularly with other lay health workers to share experiences and give each other support [26]. (See Paper 7 on supervision for further discussion of these points [64].)

A well-functioning referral chain is often vital, as is discussed in Paper 9 in this series [46]. Some tasks are given to CHWs on the condition that they are trained to recognize symptoms or danger signs and refer patients to the appropriate health facilities. Such tasks require that the nearest health facility be sufficiently staffed and equipped, that CHWs have practical ways of contacting facilities, that a collaborative relationship exists between the CHWs and the facility staff, and that recipients are willing to travel to these facilities and have the funds and the means of transport to do so. However, these factors are not always in place [26]. Both CHWs and recipients may have poor relationships with facility staff or may lack the practical means to contact them. In addition, facilities are often under-resourced, and facility staff may feel that CHW programmes will increase their workload as a result of supervision requirements or an increase in referrals, or may fear a loss of authority [26]. Health professionals may be more likely to accept CHW tasks if boundaries are clear and if they feel that the CHWs make sense in their setting (e.g., by easing some of their own busy workload). For these reasons, health professionals and their organizations need to be involved when deciding on the roles and tasks of the CHW.

CHWs may also need access to supplies. Unreliable access to necessary supplies can threaten the implementation of relatively simple interventions and lead to loss of respect in the community for the CHW and the health system [60]. Important considerations include the extent to which supplies can be stored over long periods of time or require specific storage conditions.

Finally, health system regulations may need to be changed to reflect changes in CHWs’ scope of practice and to ensure legal protection if, as rarely happens, a complication arise. A recent study on task shifting among nurses and midwives in 13 African countries suggested that many of the countries had not revised their national regulations to incorporate additional professional roles and responsibilities associated with task shifting, and this negatively the long-term sustainability of their roles [65]. Similarly, a lack of regulatory support may impede institutionalization of changes, which may also be an issue for CHW programmes. This is especially important in public health emergency contexts, where established emergency protocols for the reorientation of CHW roles can enhance the adaptive capacity of the health system. Brazil’s CHW work process in the context of COVID-19 highlights a reorganization of CHW roles to support multi-professional health teams, promote telehealth, and further expand their role as health educators [49].

### How much will it cost to use CHWs to perform the task?

Finally, programme planners need to consider the cost of using CHWs to perform specific tasks. There may be an assumption that the use of CHWs is cheaper than the use of other health worker cadres, but this is not necessarily true. Programme planners need to consider a number of potential costs:

- Training, including the costs of initial and refresher training, trainer salaries, training materials, and travel and refreshments. Decentralized, local training (possibly associated with online instruction) can potentially reduce these costs, which otherwise can be quite substantial.
- Supervision and support, including the costs of salaries for supervisors, transport and refreshments for supervisors making field visits, and replacing health workers that are being moved from other tasks to provide supervision. Regular, easily accessible supervision and support from health system and community structures could potentially also save costs by increasing retention of CHWs.
- Transport, including the costs of transport for CHWs visiting clients, accompanying clients to health facilities, and travelling to health facilities to receive supervision and deliver reports.
- Wages and other incentives, including the costs of salaries and other monetary incentives, such as lunch money, health insurance, and educational stipends. Many programmes also make use of nonmonetary incentives, such as bicycles and T-shirts. Formal recognition from the community and the health system may also be an important incentive to the CHW and may incur costs. For example, the Nepalese government has attempted to incentivize volunteer CHWs through the production of CHW stamps and postcards, an annual CHW celebration, and the production of a TV drama about the valuable contributions of CHWs [66]. However, decisions regarding appropriate incentives is context-specific and needs broad consultation and careful consideration.
- Equipment and supplies, including the costs of medical supplies and promotional materials, bicycles, uniforms, telephones, bags, and signboards. These may not all be necessary items for the provision of specific tasks, but may serve as important motivating incentives to the CHW and may increase their social status and visibility in the community.
- Referral systems, including any additional costs to the health system to enable CHW referral, including transportation systems, communication systems, and staffing of facilities. Deployment of CHWs may increase the number of referrals arising from communities, which also will have cost implications.

## Conclusions

Involving CHWs in the codesign of new roles and tasks on the basis of experiences can be a powerful approach to both empowering CHWs and to producing more effective CHWs [67]. Iterative codesign approaches can also empower CHWs to exercise discretion in role execution. As community intermediaries, CHWs can be uniquely positioned to deliver person-centred care based on the client’s care needs, cooperation, availability of a social network, and the reliability and resilience of the client’s social support system [68]. This is helpful for both intrinsic motivation in carrying out roles as well as for the health impact for clients. The person-centred approach would also help to build trust between CHWs and community members in contexts where CHWs are mistrusted, for instance due to their representation of government programmes. In addressing challenges around heavy workloads, approaches that integrate vertically oriented programmes have been successful.

Decisions regarding CHW roles and tasks are complex, and each decision has implications for the effectiveness, acceptability, feasibility, and costs of a CHW programme. Decision-makers should draw from global guidance and research evidence, but they also need to engage with and understand the experiences, needs, and concerns of local communities and health workers. By drawing from these important sources of information, planners, programme implementers, and policy-makers will stand a better chance of developing CHW programmes that are effective in achieving their goals while remaining acceptable to those affected by them, feasible to implement, sustainable over time, and sufficiently flexible and resilient to meet new challenges when they arise.

## Data Availability

Not applicable.

## Abbreviations

ASHA: Accredited social health activist
ASM: *Animatrice de Santé Maternelle*
CHW: Community health worker
COVID: Coronavirus disease
HEW: Health extension worker
iCCM: Integrated community case management (of childhood illness)
TB: Tuberculosis
